# Correction: July effect in hospitalized cirrhosis patients: A US nationwide study using difference-in-differences analysis

**DOI:** 10.1371/journal.pone.0323476

**Published:** 2025-04-30

**Authors:** Melis Gokce Celdir, George Wehby, Shahana Prakash, Tomohiro Tanaka

The Funding statement for this article is incorrect. The correct Funding statement is as follows: This work was sponsored by the Bruce L. Miller & M. Joyce Miller Memorial & Visiting Professorship for the publication fee (Awarded to MGC).
