# Supplementary material for: July effect in hospitalized cirrhosis patients: A US nationwide study using difference-in-differences analysis
Source: PLoS One. 2025 Jan 13;20(1):e0316445. doi: 10.1371/journal.pone.0316445 (PMC11729967; doi:10.1371/journal.pone.0316445)
Supplement: S1 Table — (DOCX) [file pone.0316445.s001.docx]

Supplemental Table 1. ICD-10 and CPT codes to identify the study population and severe cirrhosis complications.

| Diagnosis | ICD-10 and CPT Code |
| --- | --- |
| Cirrhosis | K70.1, K70.3, K74.60, K74* |
| Cardiovascular failure | 4A143B0, 4A133B1, R57. 9, R57. 0, R57. 1, R65.20 |
| Cerebral failure | K72.91, K70.41, G93. 82 |
| Respiratory failure | 5A1935Z, 5A1945Z, 5A1955Z |
| Renal failure | N17.0, N17.1, N17.2, N17.8, N17.9, N19, 5A1D90Z |
| Variceal bleeding | I85.01, I86.4, I85.01, I85.11, 43244 |
| Hepatorenal syndrome | K76.7 |
