# Supplementary material for: July effect in hospitalized cirrhosis patients: A US nationwide study using difference-in-differences analysis
Source: PLoS One. 2025 Jan 13;20(1):e0316445. doi: 10.1371/journal.pone.0316445 (PMC11729967; doi:10.1371/journal.pone.0316445)
Supplement: S2 Table — (DOCX) [file pone.0316445.s002.docx]

Supplemental Table 2. Inpatient mortality among patients admitted to teaching and non-teaching hospitals, according to month.

|  | Mortality in the total cohort | | | | Mortality among severe complications group | | | |
| --- | --- | --- | --- | --- | --- | --- | --- | --- |
| Month | Teaching | Non-Teaching | Adjusted Odds Ratio (95% CI)^1^ | P Value of Adjusted Odds Ratio Compared with May* | Teaching | Non-Teaching | Adjusted Odds Ratio (95% CI) ^1^ | P Value of Adjusted Odds Ratio Compared with May* |
| January | 6.2% | 5.9% | 1.08 (0.98-1.19) | 0.28 | 22.5% | 22.8% | 1.03 (0.90-1.18) | 0.06 |
| February | 5.9% | 5% | 1.18 (1.07-1.31) | 0.80 | 21.6% | 19.5% | 1.08 (0.94-1.26) | 0.36 |
| March | 5.5% | 5.3% | 1.08 (0.98-1.19) | 0.28 | 20.2% | 19.5% | 1.08 (0.94-1.25) | 0.27 |
| April | 5.5% | 5% | 1.13 (1.02-1.25) | 0.71 | 21% | 19.8% | 1.04 (0.90-1.20) | 0.20 |
| May | 5.6% | 4.9% | 1.18 (1.07-1.31) | - | 21.5% | 18.8% | 1.23 (1.06-1.42) | - |
| June | 5.4% | 4.4% | 1.26 (1.31-1.40) | 0.37 | 20.9% | 18.4% | 1.19 (1.02-1.38) | 0.83 |
| July | 5.5% | 4.7% | 1.18 (1.07-1.31) | 0.88 | 21.8% | 20.8% | 1.07 (0.93-1.23) | 0.18 |
| August | 5.4% | 4.7% | 1.18 (1.07-1.30) | 0.88 | 21.6% | 18.8% | 1.22 (1.05-1.41) | 0.95 |
| September | 5.3% | 5% | 1.05 (0.95-1.16) | 0.20 | 20.9% | 20.1% | 1.04 (0.90-1.21) | 0.20 |
| October | 5.5% | 5% | 1.11 (1.00-1.23) | 0.75 | 20.8% | 19.7% | 1.07 (0.93-1.24) | 0.41 |
| November | 5.9% | 5.1% | 1.16 (1.05-1.28) | 0.99 | 21.9% | 19.6% | 1.21 (0.97-1.29) | 0.74 |
| December | 6.3% | 5.7% | 1.13 (1.03-1.25) | 0.60 | 22.8% | 21% | 1.14 (1.00-1.31) | 0.56 |
