# Supplementary material for: July effect in hospitalized cirrhosis patients: A US nationwide study using difference-in-differences analysis
Source: PLoS One. 2025 Jan 13;20(1):e0316445. doi: 10.1371/journal.pone.0316445 (PMC11729967; doi:10.1371/journal.pone.0316445)
Supplement: S3 Table — (DOCX) [file pone.0316445.s003.docx]

Supplemental Table 3. LOS among patients admitted to teaching and non-teaching hospitals, according to month.

|  | LOS in the total cohort | | | | LOS among severe complications group | | | |
| --- | --- | --- | --- | --- | --- | --- | --- | --- |
| Month | Teaching, median days [IQR] | Non-Teaching, median days [IQR] | Adjusted Rate Ratio (95% CI) ^1^ | P Value of Adjusted Rate Ratio Compared with May* | Teaching, median days [IQR] | Non-Teaching, median days [IQR] | Adjusted Rate Ratio (95% CI) ^1^ | P Value of Adjusted Rate Ratio Compared with May* |
| January | 4 [3, 8] | 4 [2, 7] | 1.13 (1.12-1.14) | 0.21 | 6 [3,12] | 5 [3,10] | 1.20 (1.18-1.22) | 0.54 |
| February | 4 [3, 8] | 4 [2, 7] | 1.12 (1.11-1.13) | 0.43 | 6 [3,11] | 5 [3,9] | 1.13 (1.10-1.15) | 0.03* |
| March | 4 [2, 7] | 4 [2, 7] | 1.13 (1.12-1.14) | 0.37 | 6 [3,12] | 5 [3,10] | 1.22 (1.19-1.24) | 0.74 |
| April | 4 [2, 7] | 4 [2, 7] | 1.13 (1.12-1.14) | 0.01 | 6 [3,11] | 5 [3,10] | 1.17 (1.15-1.20) | 0.73 |
| May | 4 [2, 7] | 4 [2, 7] | 1.12 (1.11-1.13) | - | 6 [3,12] | 5 [3,10] | 1.19 (1.16-1.21) | - |
| June | 4 [2, 7] | 4 [2, 6] | 1.12 (1.11-1.13) | 0.15 | 6 [3,11] | 5 [3,9] | 1.18 (1.15-1.20) | <0.01 |
| July | 4 [2, 7] | 4 [2, 6] | 1.15 (1.14-1.16) | <0.01 | 6 [3,12] | 5 [3,9] | 1.29 (1.26-1.31) | <0.01 |
| August | 4 [2, 7] | 4 [2, 7] | 1.16 (1.15-1.17) | <0.01 | 6 [3,12] | 5 [3,9] | 1.23 (1.20-1.25) | <0.01 |
| September | 4 [2, 7] | 4 [2, 7] | 1.10 (1.09-1.11) | 0.11 | 6 [3,11] | 5 [3,9] | 1.23 (1.20-1.26) | <0.01 |
| October | 4 [2, 8] | 4 [2, 7] | 1.14 (1.13-1.15) | <0.01 | 6 [3,11] | 5 [3,9] | 1.24 (1.21-1.26) | <0.01 |
| November | 4 [2, 8] | 4 [2, 7] | 1.12 (1.11-1.13) | 0.11 | 6 [3,12] | 5 [3,9] | 1.16 (1.13-1.18) | 0.17 |
| December | 4 [2, 7] | 4 [2, 7] | 1.15 (1.14-1.16) | <0.01 | 6 [3,11] | 5 [3,9] | 1.24 (1.21-1.26) | 0.01 |

^1^Comparison of mortality and LOS in the respective months relative to May according to hospital type, regression analyses were run separately for each month pair. Models are adjusted for sex, age, race, hospital ownership, census division, bed size, Cirrhosis Comorbidity Score.

*p-value of the interaction term in the form of (Month)*(Teaching) with an indicator variable for each month and hospital teaching status from the fitted adjusted logistic and Poisson regression models for mortality and length of stay, respectively. Models are adjusted for sex, age, race, hospital ownership, census division, bed size, Cirrhosis Comorbidity Score.
